# Numerical and Experimental Investigation of Mixing Enhancement in a Zigzag Passive Micromixer with D-Shaped Obstacles

**DOI:** 10.3390/mi17020190

**Published:** 2026-01-30

**Authors:** Bingyang Yuan, Shuai Yuan, Hao Wang

**Affiliations:** 1 College of Shipbuilding Engineering, Harbin Engineering University, Harbin 150001, China; yuanby@hrbeu.edu.cn; 2 School of Health and Life Sciences, University of Health and Rehabilitation Sciences, Qingdao 266113, China; yuanshuai1006@hotmail.com; 3 Ocean Decade International Cooperation Center (ODCC), Qingdao 266520, China; 4 Qingdao Innovation and Development Base, Harbin Engineering University, Qingdao 266000, China; 5 Nanhai Innovation and Development Base, Harbin Engineering University, Sanya 572024, China

**Keywords:** microfluidic, Zigzag passive mixer, D-shaped obstacle, performance index

## Abstract

Micromixers are crucial for rapid and homogeneous mixing in lab-on-a-chip systems. This study presents a novel passive micromixer that synergistically combines a Zigzag channel with D-shaped obstacles to enhance mixing across a broad Reynolds number (Re) range of 0.1–50. The design leverages flow splitting, recombination, and the generation of localized high-velocity streams to effectively disrupt laminar flow. A comprehensive parametric study optimized key geometric parameters, including obstacle dimensions (b_2_, a_2_) and the number of mixing units (n). Results show that optimizing b_2_ to 500 μm and increasing a_2_ to 250 μm significantly enhances mixing efficiency. Mechanistic analysis reveals that centrifugal forces in the Zigzag channel work synergistically with obstacle-induced perturbations to stretch and fold the fluid interface, promoting transverse transport. The optimized mixer, fabricated and experimentally validated, achieves a high mixing index (>0.85) under all Re conditions. This work provides valuable design insights for developing efficient, compact micromixers for micro-total analysis systems.

## 1. Introduction

Lab-on-a-chip (LOC) technology has garnered significant research interest owing to its distinctive capabilities, including minimal sample volume requirements, rapid analysis speeds, and highly integrated miniaturization [1,2,3]. Within these integrated systems, micromixers function as essential components for reagent manipulation, finding broad applications across biological engineering [4,5], chemical detection [6,7], and synthesis processes [8]. The efficacy is critically dependent on the mixing efficiency of these micromixers. However, achieving efficient fluid mixing at the microscale is inherently challenging; the confinement to miniature dimensions, in contrast to macroscopic reactors, constrain fluid dynamics, often resulting in laminar flow regimes characterized by low Reynolds numbers. Under these conditions, where turbulent mixing is absent, molecular diffusion becomes the dominant mechanism for mass transfer [8].

Enhancing micromixing process can be achieved by increasing the diffusion coefficient, the concentration gradient, or the interfacial contact area across which diffusion occurs. Among these factors, augmenting the contact area to reduce the diffusion path length is widely regarded as the most effective strategy for improving mixing quality in microfluidic environments [9]. To address this, developed micromixers are generally categorized into two classes based on their operational principles: active mixers [10], which utilize external energy fields (e.g., acoustic, magnetic and electric [11,12,13,14]); and passive mixers [15], which rely solely on channel geometry [16,17] to induce enhanced advective stretching and folding of the fluid interface [18,19] or lamination. Passive micromixers are often favored for their straightforward fabrication, simple architecture, and ease of integration into microfluidic systems. The commonly used passive mixing modes include obstacle unit insertion [20,21], serpentine channels [22], and layered methods [23], etc.

Among passive micromixers, the serpentine microchannel is recognized as a particularly straightforward yet effective design [24]. Strategic modulation of the channel curvature radius has been identified as a highly effective method for enhancing mixing performance by actively promoting transverse secondary flow structures, notably Dean vortices [25]. These vortices arise from centrifugal in-stabilities as the fluid negotiates a serpentine path, inducing enhanced advective stretching and folding of the fluid interface that significantly augments interfacial stretching and folding, thereby reducing the diffusion path length and accelerating homogenization. For instance, Elmas et al. [26] realized online monitoring of swimming pool water quality using a simply configured serpentine microchannel. By mixing water with methyl orange and phenol red reagents, their system enabled concurrent detection of chlorine content and pH value. Hossain and Kim [27] introduced a non-aligned inlet configuration based on a conventional square-wave channel, which enhanced mixing but also increased the pressure drop. Javaid et al. [28] developed a serpentine mixer with sinusoidal sidewalls; when the amplitude-to-wavelength ratio exceeded 0.15, mixing performance surpassed that of square-wave mixers, though at a higher mixing cost under Re = 50. In another effort, Chen and Li [29] incorporated barriers into serpentine channels to induce vortex flow and applied topology optimization to determine the best geometry. The optimized mixer exhibited superior mixing to a conventional Zigzag mixer.

Integrating obstacles into traditional serpentine micromixers can destabilize the primary flow and shift the centers of vortex rotation, generating complex, asymmetrical flow fields conducive to mixing even at low Reynolds numbers where laminar flow dominates and molecular diffusion is inherently slow. This geometric strategy successfully overcomes the reliance on high flow rates or extended channel lengths for efficient mixing, making such designs particularly suitable for LOC applications requiring rapid and efficient reagent mixing within compact footprints. Mondal et al. [30] examined mixing enhancement in a square-wave serpentine micromixer by integrating wall obstacles. Compared to a plain mixer, all obstacles induced enhanced advective stretching and folding of the fluid interface, reducing the required mixing length by 30%. Significantly, the triangular obstacles achieved the optimal trade-off, delivering highly efficient mixing while incurring the lowest pressure drop among the designs. Mirkarimi et al. [31] investigated the mixing enhancement in a curved serpentine micromixer by incorporating cylindrical obstacles in some distinct arrangements. The performance was compared against a simple curved channel across Reynolds numbers (Re) from 0.1 to 80. Results demonstrated that at low Re, obstacles significantly improved mixing efficiency by inducing flow perturbations and increasing interfacial contact area. However, at high Re, the unobstructed channel outperformed others as obstacles hindered the development of centrifugal force-driven secondary flows. Tan et al. [32] developed a novel 3D homogeneous microreactor by integrating obstructive structures into a Zigzag microchannel to significantly enhance mixing performance across a wide Re range. The optimized obstructions generated multi-directional secondary flows, inducing enhanced advective stretching and folding of the fluid interface that drastically reduced both mixing distance and time.

Despite the significant progress made in the above research, this field still faces ongoing challenges, especially the lack of efficient mixing within a wide range in planar microchannels. This study aims to maximize the promoting effect of secondary flow structures on micromixing under low Re conditions and proposes a design that incorporates secondary flow structures to enhance mixing efficiency by reducing the mixing length and mixing quality. This design can disturb the stable laminar flow phenomenon to the greatest extent and increase the fluid contact area to enhance the molecular diffusion strength.

## 2. Micromixer Geometry Model

### Physical Model

**Figure 1:**
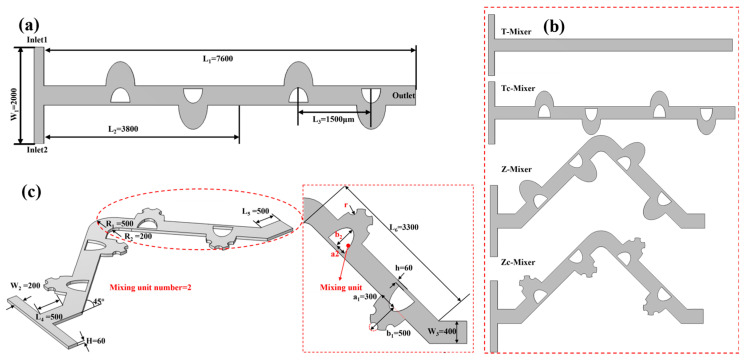
Illustration of micromixer with two mixing units: (**a**) the geometry of T-mixer, (**b**) the geometry of T-type and Zigzag-type mixers; (**c**) the geometry of Zc-mixer. The unit in these figures is μm.

The numerical simulations in this study are based on the four passive planar micromixer configurations illustrated in Figure 1: T-Mixer, Tc-Mixer, Z-Mixer, and Zc-Mixer. In the early stage of numerical simulation, all configurations incorporate two identical mixing units, the core geometric features of which are detailed in Figure 1a–c.

## 3. Research Methods

### 3.1. Numerical Simulation Methods and Theoretical Formulas

The flow field and mixing performance were simulated using COMSOL Multiphysics^®^ (v6.1) based on the Finite Element Method (FEM). A segregated, two-step approach was employed: First, the steady-state velocity and pressure fields were obtained by solving the Navier–Stokes equations using the “Laminar Flow” interface. Second, this converged velocity field was used as input to solve the steady-state convection-diffusion equation for species concentration using the “Transport of Diluted Species” interface. All solutions were considered converged when the scaled residuals were below 1 × 10^−6^.

The governing equations for the incompressible Newtonian fluid are as follows [33]:

(1) $$\nabla \times \vec{U}=0,$$

(2) $$\rho \left(\vec{U}\cdot \nabla \right)\vec{U}+\nabla P-\mu \nabla^{2}\vec{U}=0,$$

(3) $$\left(\vec{U}\cdot \nabla \xi \right)=D\nabla^{2}\xi ,$$

where $\vec{U}$, ρ, P, D, μ and ξ stand for the fluid velocity vector, the fluid density, the pressure, the diffusion coefficient, the absolute viscosity and the specie concentration, respectively. The numerical calculations were carried out in two steps: first, Equations (1) and (2) were used to obtain the result of velocity field, which was substituted into Equation (3). Then, the concentration distribution was solved.

To numerically evaluate the mixing performance, two working fluids were injected into the micromixer through its two inlets (Inlet 1 and Inlet 2, see Figure 1). The relevant physical properties of these fluids are listed in Table 1. The boundary conditions were defined as follows. Both inlets were set as specified-velocity inlets with equal flow rates to establish symmetric feeding. The species concentration was prescribed as 0 mol·m^−3^ for Inlet 1 and 1 mol·m^−3^ for Inlet 2. Note that a normalized concentration scalar (ξ) is solved, where ξ = 0 and 1 at the inlets represent the pure components. This normalized field is used for calculating the mixing index and directly corresponds to the experimental fluids: ξ = 0 maps to deionized water and ξ = 1 maps to the 0.5 mol/L methyl blue solution. The molecular diffusion coefficient is set to D = 1 × 10^−9^ m^2^/s, a representative value for such dye molecules in aqueous solutions at room temperature. The outlet was assigned a pressure-outlet condition with zero static pressure (0 Pa gauge). All channel walls were treated as no-slip boundaries for flow, and for species transport a zero-normal-flux (insulating) condition was applied at the walls, consistent with the assumption of passive mixing without surface reactions.

An essential dimensionless parameter for characterizing flow regimes under microscale conditions is the Reynolds number (Re), defined as the ratio of inertial forces to viscous forces acting on the fluid [34]:

(4) Re=ρUDhμ

where D_h_ is the hydraulic diameter of microchannel at the outlet.

The mixing quality is evaluated by the mixing index (M.I) based on the concentration variance at the outlet cross-section, defined as [35]:

(5) $$Μ.Ι=1-\frac{\sigma_{m}}{\sigma_{m,\max}},$$

(6) $$\sigma_{m}=\sqrt{\frac{1}{m}\sum_{k=1}^{m}\left(\xi_{k}-\bar{\xi }\right)}$$

where $\sigma_{m}$ is the concentration variance, ξ_k_ is the concentration value at the sampling point, and $\bar{\xi }$ is the average component concentration, and m is the total number of sampling points. The normalization factor σ_m,max_ is the variance of the completely unmixed state, i.e., a step concentration profile of 0 and 1 across the channel inlet. To ensure consistency, σ_m,max_ is calculated using the same sampling grid as the actual flow field. In this study, m = 200 sampling points were placed uniformly along a straight line passing through the center of the outlet channel. This sampling protocol was applied identically to all simulation cases and to the analysis of experimental images. For the experiments, the grayscale intensity from the captured images was normalized to obtain the concentration field, and the M.I was computed along the corresponding centerline using the same sampling logic, enabling a direct and fair comparison with the numerical results. By definition, M.I = 1 indicates perfect mixing, and M.I = 0 indicates complete segregation.

The hydraulic characteristic of the micromixer was evaluated based on the pressure drop (ΔP) measured between the inlet and outlet.

(7) $$\Delta P=P_{\mathrm{inlet}}-P_{\mathrm{outlet}},$$

where P_inlet_ and P_outlet_ are the mean pressure at the inlet and outlet of the miromixer.

To comprehensively assess the micromixer performance over a wide Re number range, a new evaluation metric termed the performance index (PFI) was introduced [36]. This index simultaneously accounts for the M.I and the ΔP inherent to the microchannel. The PF is defined as follows:

(8) $$PF=\frac{M.I}{\Delta P},$$

The dimensionless performance factor (PF) defined in Equation (8) was normalized for each case according to Equation (9) to obtain the normalized performance factor (NPF):

(9) $$NPF=\frac{PF}{PF_{max}},$$

where PF_max_ is the maximum value of PF in each condition.

Finally, the mean value of the NPF was computed by averaging its values over the entire Re spectrum, as formulated in Equation (10). It is evident that a higher performance index corresponds to enhanced overall performance throughout the Re range.

(10) $$PFI=\frac{\sum_{1}^{n}NPF}{n},$$

where n is the total number of Re cases.

The PFI, calculated as the mean of NPF values over the entire Re spectrum, provides a comprehensive metric to evaluate and compare the overall efficiency of different mixer designs across their operating range, mitigating bias from performance variations at specific Reynolds numbers.

### 3.2. Mesh Independence Verification

A comprehensive mesh sensitivity analysis was performed to ensure the numerical results were independent of the spatial discretization across the Reynolds number range studied. Given the importance of accurately resolving both the flow field and the concentration gradients—especially in the narrow gaps and near obstacle walls—five mesh schemes with progressive refinement were generated and evaluated at the challenging, diffusion-dominated condition of Re = 5. The key parameters of these meshes and the resulting outlet pressure drop and mixing index are summarized in Table 2. An explicit convergence criterion was defined: a change of less than 1% in ΔP and less than 3% in M.I between successive refinements is considered acceptable for establishing mesh independence for practical engineering analysis. It should be noted that at Re = 50, mesh scheme 4 also demonstrated convergence, with detailed mesh information provided in Sporting Material Table S1.

A comprehensive mesh sensitivity analysis was performed to ensure the numerical results were independent of the spatial discretization across the Reynolds number range studied. Given the importance of accurately resolving both the flow field and the concentration gradients—especially in the narrow gaps and near obstacle walls—five mesh schemes with progressive refinement were generated and evaluated at the challenging, diffusion-dominated condition of Re = 5. The key parameters of these meshes and the resulting outlet pressure drop and mixing index are summarized in Table 2. An explicit convergence criterion was defined: a change of less than 1% in ΔP and less than 3% in M.I between successive refinements is considered acceptable for establishing mesh independence for practical engineering analysis. To confirm the robustness of the chosen mesh across flow regimes, a separate verification at the higher, advection-influenced condition of Re = 50 was also conducted. The results, including mesh parameters, calculated ΔP and M.I, and a description of the local refinement applied, are provided in the Supplementary Material (Table S1). This analysis confirms that Mesh scheme 4 also achieves convergence at Re = 50.

### 3.3. Chip Fabrication and Experiment Apparatus

**Figure 2:**
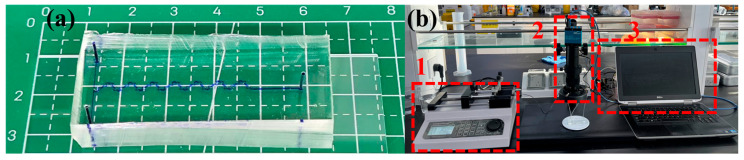
The experiment setup for exploring mixing mechanism: (**a**) microfluidic chip; (**b**) The experiment setup: 1 is a syringe pump; 2 is an electron microscope with a camera; 3 is a computer.

The silicon master mold was fabricated using standard soft lithography techniques [37]. The mask pattern for the microchip was first designed in AutoCAD (2020) software and subsequently printed onto a flexible transparent plastic film using a laser printer with a resolution of 25,400 dpi. Microstructural molds were fabricated on silicon wafers using the negative photoresist SU8-3035 (SU-8 3035, MicroChem Corp., Newton, MA, USA) via standard soft lithography procedures. Subsequently, the PDMS prepolymer (PRT-615, Momentive Corp., Waterford, NY, USA) and the cross-linker were mixed at a mass ratio of 10:1. The mixture was degassed for 30 min in a mechanical vacuum chamber to eliminate air bubbles. The uncured mixture was then poured onto the silicon mold and cured at 100 °C for 2.5 h. Following this, the PDMS layer containing the replicated microchannels was carefully peeled off from the master mold, and inlet/outlet holes were vertically punched on the slab. Finally, the PDMS layer was permanently bonded to a clean glass substrate to form a sealed PDMS/glass microdevice. This bonding process was achieved using a plasma cleaner (PDC-002, Harrick, Ithaca, NY, USA) operating at 160 W for 100 s, followed by heating at 100 °C for 2.5 h. The manufactured chip is shown in Figure 2a.

To validate the internal fluid flow and mixing performance of the micromixer, the experimental setup established in this work is illustrated in Figure 2b. The key apparatus includes an electron microscope with a camera (Chenxi CX-H5000, Shenzhen, Guangdong, China), syringe pumps (Longer LSP04-1A, Baoding, Hebei, China). Two different working fluids—deionized water and an aqueous methyl blue solution (0.5 mol/L)—were infused into the micromixer via the pumps, with flow rates ranging from 0.72 μL/min to 360 μL/min, corresponding to a Re number range of 0.1 to 50. During the experiments, the camera captured images along each segment of the mixing channel. The mixing performance at various Re values was experimentally obtained by extracting grayscale values from the images using ImageJ software(v1.53t), where the grayscale value at the target cross-section was used to represent concentration. The final mixing index was calculated according to Equations (5) and (6).

## 4. Results and Discussion

### 4.1. Effects of Channel Type on Mixer Performance

**Figure 3:**
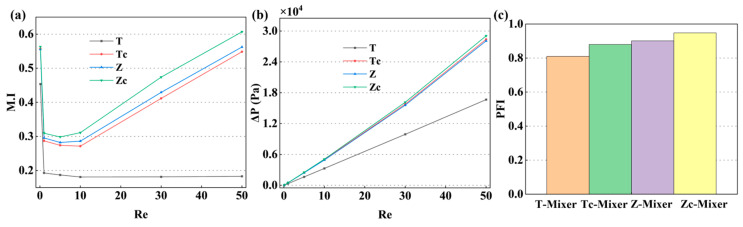
Effects of channel type on micromixer performance when b2 = 500 μm and a2 = 200 μm: (**a**) M.I; (**b**) ΔP; (**c**) PFI.

The simulation results revealed the significant influence of micromixer channel shape and mixing units on the M.I and PFI, providing a theoretical basis for determining the optimal microreactor configuration that combines high mixing efficiency with an acceptable pressure drop. To investigate the influence of the key dimensions of the mixing unit, we first analyzed the performance of four micromixers (their structures are shown in Figure 1b) under the condition of mixing unit dimension b_2_ = 500 μm and a_2_ = 200 μm. As the results in Figure 3a show, the M.I of all configurations exhibited a common trend of first decreasing and then increasing with the increase of Re. It is noteworthy that this macroscopic trend was independent of the basic channel configuration (T-type or Z-type) and the presence of D-shaped obstacles, suggesting that the transition in flow state was the dominant factor within this Re range. However, the absolute value of mixing efficiency was strongly dependent on the specific geometric design. At Re = 10 (corresponding to the M.I trough), the basic T-Mixer exhibited the weakest mixing effect, with an M.I of 0.18. Incorporating a D-shaped obstacle into the T-channel to form the Tc-Mixer increased the M.I to 0.27. Changing the channel to a Zigzag shape (Z-Mixer) yielded a similar improvement, achieving an M.I of 0.28. The Zc-Mixer, which combines the Zigzag channel with the D-shaped obstacle, demonstrated optimal performance, with an M.I of 0.30. These results indicate that both optimizing the basic channel configuration and introducing internal mixing units effectively enhanced mixing, exhibiting a synergistic effect between the two approaches. Further analysis of the ΔP reveals distinct hydraulic characteristics among the configurations. The T-Mixer exhibits the lowest ΔP, establishing it as the baseline reference. The introduction of a D-shaped obstacle in the Tc-Mixer increases local flow resistance, resulting in an 8.6% rise in ΔP compared to the baseline, as shown in Figure 3b. The Zigzag of the Z-Mixer further elevates the ΔP due to prolonged flow path and frequent directional changes that intensify energy dissipation. Consequently, the Zc-Mixer, which integrates both the Zigzag channel and the D-shaped obstacle, records the maximum ΔP. Nevertheless, this value remains within an acceptable range for practical engineering applications. Therefore, considering the practical constraint of an acceptable pressure drop, the Zc-Mixer emerges as the optimal choice due to its attainment of the highest PFI, as shown in Figure 3c.

**Figure 4:**
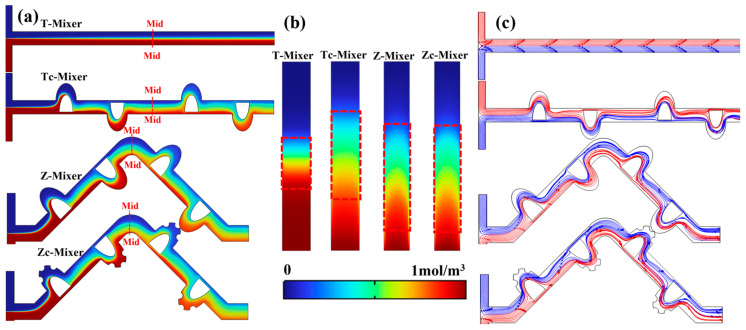
The influence of microchannel types on concentration and flow field distribution when Re = 50: (**a**) the concentration variation in different channels; (**b**) concentration distribution in the middle of the channel (Mid-Mid); (**c**) the velocity variation in different channels.

To elucidate the underlying mechanisms responsible for the observed performance discrepancies, a detailed analysis was conducted on the mixing enhancement mechanisms. This analysis combined the corresponding concentration and flow field distributions under the given operating conditions. As illustrated in Figure 4a,c, the T-Mixer sustains a stable laminar flow regime within its channel, characterized by parallel streamlines and minimal interfacial interaction. Consequently, mixing relies solely on inefficient molecular diffusion, resulting in the lowest M.I. To overcome this limitation, the Tc-Mixer integrates a D-type micromixing unit. This design forces the fluid to undergo a “split-and-recombine” process and generates micro-jets as the fluid passes through narrow gaps between obstacles and walls. These mechanisms collectively shift the dominant mixing regime from diffusion-controlled to enhanced advective stretching and folding of the fluid interface. The concentration and velocity field distributions at Re = 50 clearly demonstrate that the mass transfer area within the Tc-Mixer is effectively doubled compared to the T-Mixer, as show in Figure 4b. This enhanced mixing efficiency leads to a significant improvement in the PFI, increasing it from 0.81 to 0.88.

To further enhance mixing efficiency, we replaced the base channel with a Zigzag geometry (Z-Mixer). In this configuration, the fluid undergoes repeated stretching and compression at the turning points due to centrifugal forces, actively disrupting the laminar interface and promoting mass transfer. However, in low-aspect-ratio microchannels, geometric redirection alone is insufficient to generate strong vortices. To address this limitation, semi-cylindrical obstacles were integrated into the mixing unit of the Zc-Mixer. These obstacles continuously perturb the flow within the cavity, significantly intensifying the enhanced advective stretching and folding of the fluid interface. Consequently, the Zc-Mixer achieves the optimal mixing performance, with an PFI value reaching up to 0.94. Based on these results, the Zc-Mixer was selected as the baseline configuration for subsequent parametric optimization studies.

### 4.2. Effect of the Obstacle Dimension b_2_ on Mixing Performance

To clarify the influence of each structural parameter, the single-variable method was employed to investigate the key geometric parameters of the obstacle, namely the major and minor axes (b_2_ and a_2_). This section systematically examined the impact of the dimension b_2_ (ranging from 300 μm to 700 μm) on the performance and underlying mechanisms of the Zc-Mixer.

**Figure 5:**
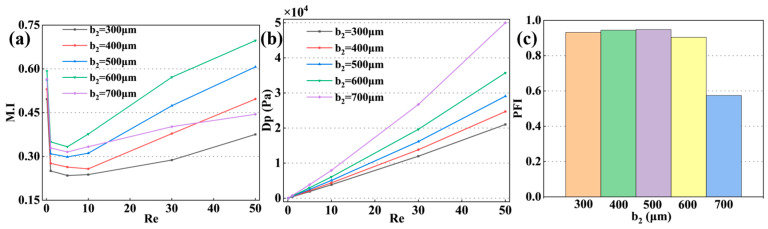
Effect of the obstacle dimension b_2_ on mixing performance. (**a**) variation of M.I with Re for different b_2_; (**b**) corresponding ΔP across the micromixer; (**c**) PFI as a function of Re for various b_2_ values.

As shown in Figure 5, the M.I for the Zc-Mixer reached its minimum value at Re = 5. When b_2_ was increased from 300 μm to 500 μm, the M.I. improved from 0.23 to 0.30, while the required driving pressure rose from 2 kPa to 2.5 kPa. Correspondingly, the PFI of the microreactor peaked at 0.9. However, a further increase in b_2_ to 600 μm resulted in a 14.9% improvement in M.I, but the ΔP increased by 22.8% compared to the case at b_2_ = 500 μm, leading to a decline in the PFI. Continuing to increase b_2_ to 700 μm caused a 36.2% reduction in M.I and a 40.0% rise in ΔP, resulting in a sharp decrease of the PFI to 0.5. These findings indicated that within a certain range, increasing the size of b_2_ enhanced both mixing performance and energy utilization efficiency. Beyond an optimal threshold, however, further enlargement led to decreased mixer performance, where more energy was dissipated to overcome flow resistance without a corresponding improvement in mixing effectiveness.

**Figure 6:**
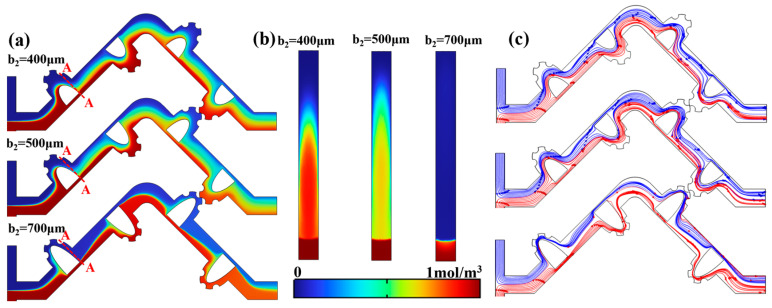
At Re = 50: (**a**) the concentration variation under different b_2_; (**b**) concentration distribution at the jet exit (A-A); (**c**) the velocity variation under different b_2_.

This non-linear performance shift was more pronounced under the working condition of Re = 50. As b_2_ increased from 300 μm to 600 μm, the mixing performance consistently improved. Analysis of the concentration and flow fields in Figure 6a,c revealed the mechanisms behind this enhancement stage: the effective volume of the mixing unit chamber decreased with increasing b_2_, intensifying fluid disturbance and mixing rate within the chamber. Simultaneously, a larger volume of fluid, disturbed by the obstacle, contributed to the formation of a stronger jet flow. The concentration distribution at cross-section A-A indicated a significantly enhanced impact intensity when the split fluids converged, with the effective mass transfer area increasing by approximately 33% and streamlines interweaving over a broader region, as shown in Figure 6b. Consequently, the mass transfer rate within the same mixing distance was markedly improved.

However, when b_2_ was increased to 700 μm, the performance deteriorated significantly. The chamber volume became excessively small, which allowed only a small portion of fluid with concentration gradients to participate in internal mixing. Although the slit between the obstacle and the chamber wall still met the conditions for jet formation, the main flow pattern evolved into two relatively stable, parallel jets after passing through the mixing unit. In this scenario, the primary function of the mixing unit shifted from enhancing mixing to accelerating the flow. Consequently, the M.I for b_2_ = 700 μm dropped substantially compared to b_2_ = 600 μm, particularly at Re = 50, decreasing from 0.70 to 0.44.

Based on a comprehensive consideration of mixing performance and the PFI index across different Reynolds numbers, b_2_ = 500 μm was identified as the optimal dimension. This size achieved the best balance between high mixing efficiency and acceptable pressure loss, and was selected as the baseline configuration for subsequent research on key structural parameter optimization.

### 4.3. Effect of the Obstacle Dimension a_2_ on Mixing Performance

**Figure 7:**
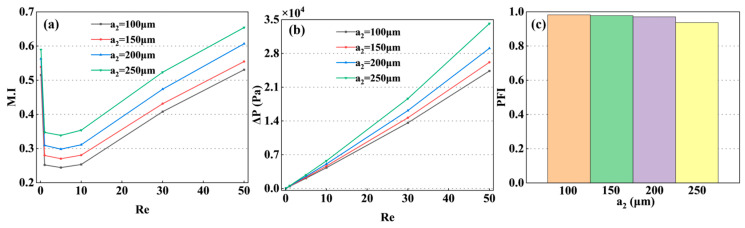
Effect of the obstacle dimension a_2_ on mixing performance: (**a**) variation of M.I with Re for different b_2_; (**b**) corresponding ΔP across the micromixer; (**c**) PFI as a function of Re for various b_2_ values.

**Figure 8:**
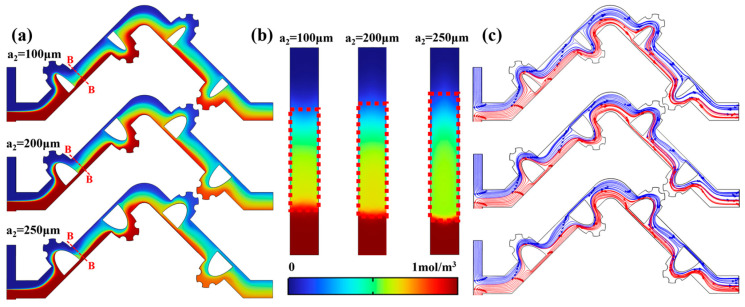
At Re = 50: (**a**) the concentration variation under different a_2_; (**b**) concentration distribution after obstacle (B-B); (**c**) the velocity variation under different a_2_.

Based on the preliminary analysis of the four basic configurations, a nonlinear relationship between mixing performance and the obstacle size within the mixing unit has been identified. To quantify this relationship, this section focuses on the critical dimension a_2_, and systematically analyzes its impact on the M.I and the PFI as it varies from 100 μm to 250 μm. As shown in Figure 7a, increasing a_2_ significantly enhances mixing efficiency at Re = 5. Specifically, when a_2_ is increased from 100 μm to 250 μm, the M.I rises from 0.24 to 0.34. This indicates that, Reconditions, a larger obstacle size effectively overcomes the diffusion-dominated mixing bottleneck by intensifying convective disturbances. However, this improvement comes at the cost of an increased ΔP, rising from 2138 Pa to 2825 Pa, which leads to a slight decrease in the PFI from 0.98 to 0.94, as shown in Figure 8b,c. At a higher Reynolds number (Re = 50), the positive effect of increasing a_2_ persists, further elevating the M.I from 0.53 to 0.65. The results synthesized from both low- and high-Re regimes a_2_ not only significantly enhances mixing performance at low Re but also broadens the effective operating range of the mixer, thereby improving its capability for rapid and uniform mixing within short channel lengths.

As illustrated in Figure 8a–c, the reduction in the effective chamber volume that accompanies an increase in a_2_ fundamentally alters the internal flow dynamics. The fluid undergoes bifurcation upon exiting the mixing unit: one portion achieves relatively thorough mixing within the constricted cavity, while the other accelerates to form a high-speed jet that impinges strongly on the downstream mainstream. The synergistic action of “cavity mixing” and “jet impingement” effectively promotes enhanced advective stretching and folding of the fluid interface. When a_2_ is increased to 250 μm, the effective mass transfer area increases by approximately 25% compared to the case of a_2_ = 100 μm, and the disturbance range is significantly expanded. Although further increasing a_2_ yields only marginal gains in M.I, the substantial rise in pressure drop causes the PFI to decline by 0.04. Therefore, by balancing mixing efficiency against energy consumption, the configuration with a_2_ = 250 μm demonstrates optimal overall performance for achieving efficient and uniform mixing over short distances. Consequently, a_2_ = 250 μm is identified as the optimal dimension in this study.

### 4.4. Effect of the Number of Cycles on Mixing Performance

**Figure 9:**
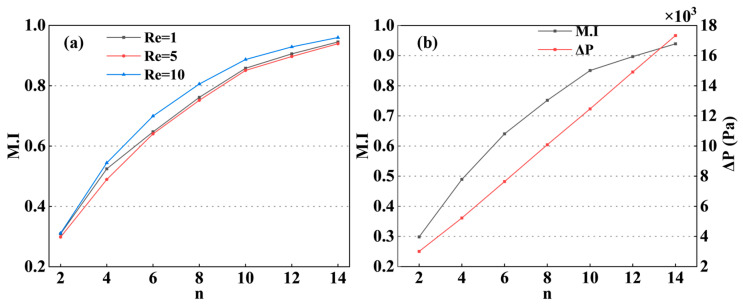
(**a**) Effect of the number of mixing units on the mixing index (Re = 1, 5, 10); (**b**) effect of the number of mixing units on ΔP (Re = 5).

While the mixing capacity of a single unit is limited, the use of multiple units in series is a common strategy to enhance mixing efficiency in micromixers. Based on the optimized configuration (a_2_ = 250 μm, b_2_ = 500 μm), the relationship between the M.I and the number of units (n) was investigated at Re = 5, where the M.I reached its minimum value in Figure 9a.

**Figure 10:**
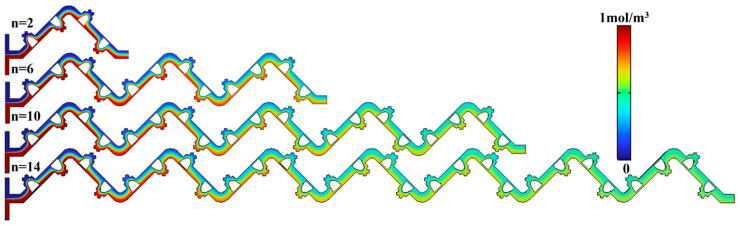
Concentration distributions with different mixing units number (Re = 5).

The M.I exhibited a characteristic nonlinear increase with n, as shown in Figure 10. As n increased, the working fluid experienced more disturbances, which enhanced the mixing quality. A rapid improvement in M.I was observed as n increased to 10, primarily due to the significant concentration gradient across the mass transfer interface that accelerated molecular transport under perturbation. However, beyond n = 10, the concentration difference gradually diminished, resulting in a reduced growth rate of the M.I. At n = 14, an M.I of 0.94 was achieved, indicating that the fluid was essentially well-mixed. This enhancement in mixing, however, came with a hydraulic cost. The ΔP increased nearly linearly with n, as shown in Figure 10. This linear increase is a direct result of the cumulative flow resistance introduced by each additional mixing unit. Therefore, although increasing n is an effective strategy to ensure complete mixing across a wide range of Reynolds numbers (particularly at low Re), a trade-off between mixing efficiency and flow resistance must be considered. Consequently, for the optimized Zc-Mixer, 14 mixing units (n = 14) were identified as the optimal configuration, as shown in Figure 10, achieving high-efficiency mixing (M.I > 0.9) at Re = 5 while maintaining an acceptable pressure drop.

### 4.5. Mechanistic Interpretation and Parametric Sensitivity Analysis

**Figure 11:**
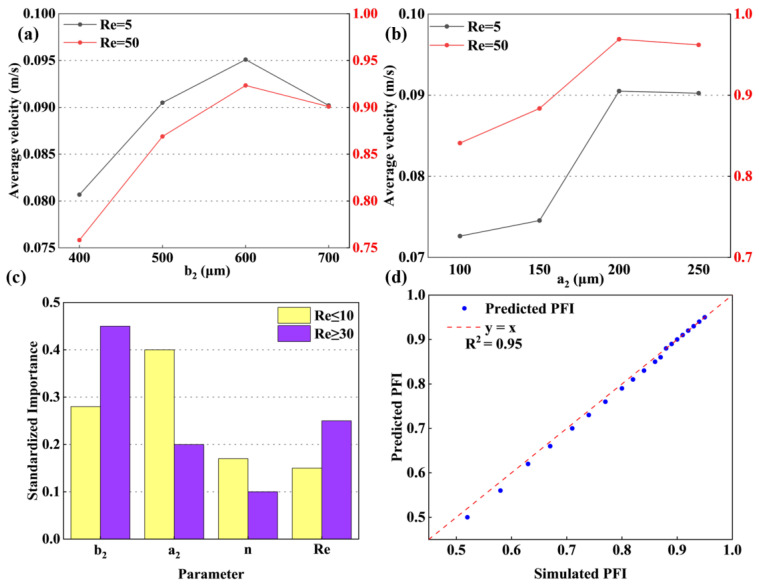
Results of parametric analysis. (**a**) Average velocity profiles at cross-section A-A for different b_2_ values at a fixed a_2_ = 250 μm. (**b**) Average velocity profiles at cross-section B-B for different a_2_ values at a fixed b_2_ = 500 μm. (**c**) Parameter importance ranking obtained from ANOVA-based regression for low- and high-Re regimes. (**d**) Comparison between the predicted PFI from the second-order response surface model and simulation results (R^2^ = 0.95).

To elucidate the underlying mechanisms through which key geometric parameters influence mixing performance, we conducted a quantitative flow field analysis to establish a direct link between parametric variations and the dominant transport phenomena. The results indicate that the obstacle width b_2_ primarily modulates the jet characteristics: a reduction in b_2_ enhances flow contraction and increases the jet velocity, as shown in Figure 11a,b. At Re = 50, the vorticity intensity downstream of the obstacle in the optimal b_2_ (500 μm) configuration is, on average, approximately 25% higher than in non-optimal cases. This quantitatively confirms its role in promoting enhanced advective stretching and folding of the fluid interface and interfacial stretching via enhanced jet momentum and impingement. The influence of the obstacle height a_2_ is more pronounced in its excitation of transverse convection. Increasing a_2_ from 200 μm to 250 μm led to an approximate 40% increase in cross-sectional secondary flow intensity, accompanied by a 35% rise in the average shear rate. This directly explains the observed trade-off between improved mixing—primarily attributed to enhanced transverse mass transfer—and increased ΔP, which stems from greater shear dissipation. Consequently, parameter optimization essentially involves identifying the optimal balance between maximizing beneficial flow disturbances and minimizing the associated flow resistance.

Building upon this clarified mechanistic understanding and to reinforce the generalizability and rigor of the optimization conclusions, we performed a systematic parametric sensitivity analysis and developed a lightweight predictive model. This approach aligns with established methodologies for performance mapping in microfluidics [38,39]. A standardized regression analysis based on Analysis of Variance (ANOVA) was employed to quantify the contribution of each parameter to the comprehensive performance metric, as shown in Figure 11c,d. As shown in Figure 11c, the importance ranking in the low-Re regime is a_2_ > b_2_ > n > Re, highlighting the effectiveness of increasing obstacle height to enhance convective disturbances under diffusion-dominated conditions. Conversely, the ranking shifts to b_2_ > Re > a_2_ > n in the high-Re regime, indicating that controlling the jet width becomes the most critical factor, while the influence of flow inertia itself is significantly amplified. This sensitivity ranking clearly delineates the shift in parametric dominance with changing flow regimes.

Utilizing the complete simulation dataset, we constructed a second-order polynomial response surface model—a lightweight surrogate—that correlates the input variables (b_2_, a_2_, and Re) with the output PFI, achieving a good fit (R^2^ = 0.94). As illustrated in Figure 11d, the model predictions show excellent agreement with the simulation results (R^2^ = 0.95). Furthermore, the generated design guidance map visually demonstrates that the identified optimal parameter combination (b_2_ = 500 μm, a_2_ = 250 μm) consistently resides within the high-performance region (PFI > 0.9) across a broad Re range from 5 to 50. This robustly confirms that the optimization outcome is not specific to a discrete set of simulated cases but remains valid over a wide operational range. Such a predictive model provides transferable quantitative guidance for the design of analogous micromixers, consistent with the growing trend of employing surrogate modeling for fluidic device optimization [40].

In summary, by integrating quantitative mechanistic interpretation with parametric sensitivity analysis and predictive modeling, this study not only reveals the physical principles governing the mixing-pressure drop trade-off in relation to geometric parameters but also substantiates the optimization findings with enhanced rigor and generalizability, thereby offering a practical and reliable design framework.

### 4.6. Experimental Verification

**Figure 12:**
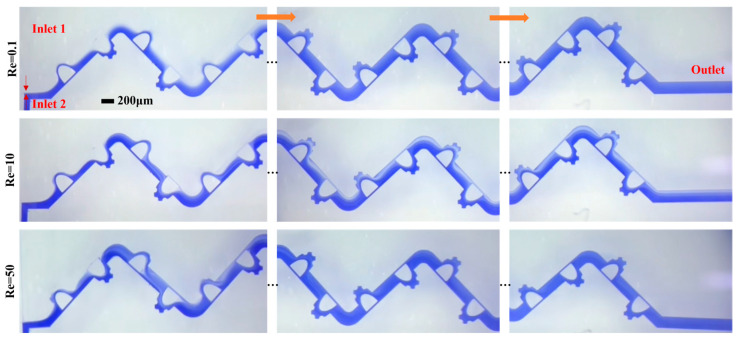
The mixing process under different Re working conditions.

**Figure 13:**
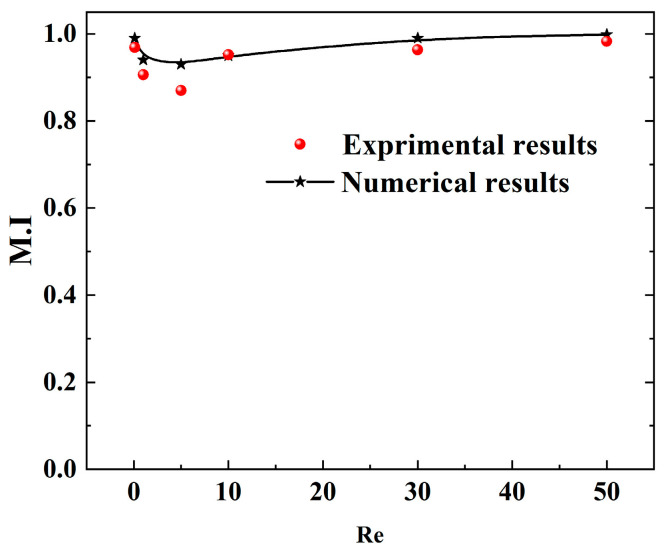
Comparison between experiments and numerical simulations.

Flow visualization was employed for both qualitative and quantitative assessment of the mixing mechanism. Figure 12 presents a comparative sequence of the mixing process captured at different flow rates. The images reveal a significant dependence of the fluid mixing state within the microchannel on the Re number. At a low Re number of 0.1, two distinct interfaces form between the different fluid streams. The flow remains laminar even after passing the Zigzag structure, confining mixing primarily to the contact interfaces where it is driven by lateral molecular diffusion. However, the significantly reduced fluid velocity allows for sufficient residence time, enabling nearly complete mixing within the micromixer. These experimental observations align with prior numerical simulations. As the Re number increases to 10, the jet-like high-velocity core stream [18,19] and secondary flow within the microchannel begin to exert a beneficial influence on fluid disturbance, with their intensity strengthening at higher Re values, thereby improving mixer performance. With a continued increase in the inlet flow up to Re = 50, the chaotic convective disturbances become more pronounced. Simultaneously, mixing in the straight channel section perpendicular to the flow direction is enhanced after the fluids pass the cross-sectional area, as the constricting effect of the jet-like high-velocity core stream gap increases the lateral velocity of the fluids, thereby expanding the mass transfer contact area and improving mixing quality at elevated Re. Across the investigated Reynolds number range, the mixing intensity in the microchannel initially decreases and subsequently increases, a trend consistent with the numerical findings. To further validate the micromixer performance, the mixing efficiency was quantified experimentally. The M.I from both experimental and simulation results, shown in Figure 13, reaches a minimum at Re = 5 before increasing to a high level at Re = 50, demonstrating good agreement between the two datasets. It can be seen that the overall mixing quality of the mixer is higher than 0.85.

To more objectively evaluate the overall performance of the proposed Zc-Mixer, a comparative analysis was conducted against several representative passive micromixers reported in the recent literature. As summarized in Table 3, the channel configurations, corresponding M.I, and Re ranges are systematically presented. It is noteworthy that the present mixer, which synergistically combines a Zigzag channel with D-shaped obstacles, achieves consistently high mixing efficiency (M.I > 0.93) across a relatively broad Re range of 0.1–50. Compared with most existing designs, the proposed structure exhibits superior mixing performance under similar or lower Reynolds number conditions, further confirming its effectiveness and structural advantages in enhancing microfluidic mixing.

## 5. Conclusions

This study systematically investigated and optimized a novel passive micromixer integrating Zigzag channels with D-shaped obstacles. Comprehensive parametric analysis identified an optimal configuration with obstacle dimensions of b_2_ = 500 μm and a_2_ = 250 μm, which delivered superior mixing performance across a wide Re range (0.1–50). The optimized design (Zc-Mixer with n = 14 units) achieved excellent mixing efficiency (M.I. > 0.93 at Re = 5) while maintaining an acceptable pressure drop. The mixing enhancement stems from the synergistic effect between centrifugal force-driven interfacial stretching and the strong flow perturbations induced by the obstacles, which together significantly increase mass transfer. Experimental results corroborated the numerical simulations, confirming the high mixing index (exceeding 0.85 at all tested Re). This work provides clear design guidelines for high-performance, compact passive micromixers, offering a practical and efficient solution for achieving rapid and uniform mixing in microfluidic applications.

## Figures and Tables

**Table 1:** Parameters of simulation experiment.

Parameter	ρ (kg/m^3^)	T (K)	μ (Pa·s)	D (m^2^/s)
Description	Density	Temperature	Viscosity	Diffusion coefficient
Values	1000	300	1 × 10^−3^	1 × 10^−9^

**Table 2:** Results of the mesh independence study at Re = 5.

Mesh Scheme	Maximum Mesh Size [μm]	Minimum Mesh Size [μm]	ΔP [Pa]	$\frac{\left|\Delta P_{k+1}-\Delta P_{k}\right|}{\Delta P_{k}}\times 100\%$	M.I	$\frac{\left|M.I_{k+1}-M.I_{k}\right|}{M.I_{k}}\times 100\%$
1	17	1.01	2521	-	0.334	-
2	15.2	0.98	2518	<0.1%	0.319	4.5%
3	13.1	0.85	2515	<0.1%	0.299	6.3%
4	10	0.65	2512	<0.1%	0.264	11.7%
5	9	0.58	2511	<0.1%	0.252	2.44%

**Table 3:** Comparison of passive mixers performance.

Reference	Channel Configuration	M.I	Re Range
[17]	straight	>0.6	0.1–100
[29]	Zigzag	>0.89	0.1–100
[30]	Square wave	1	1, 5, 10
[31]	Serpentine	>0.89	0.1–80
This work	Zigzag	>0.93	0.1–50

## Data Availability

The original contributions presented in this study are included in the article. Further inquiries can be directed to the corresponding author.

## Supplementary Materials

The following supporting information can be downloaded at: https://www.mdpi.com/article/10.3390/mi17020190/s1, Table S1. Results of the mesh independence study at Re = 50.
