# Supplementary material for: Numerical and Experimental Investigation of Mixing Enhancement in a Zigzag Passive Micromixer with D-Shaped Obstacles
Source: Micromachines (Basel). 2026 Jan 30;17(2):190. doi: 10.3390/mi17020190 (PMC12943698; doi:10.3390/mi17020190)
Supplement: Supplementary file 1 [file micromachines-17-00190-s001.zip › micromachines-4099808-supplementary.pdf]

Supplementary Materials

Numerical and Experimental Investigation of Mixing Enhancement in a Zigzag Passive Micromixer with D-Shaped Obstacles

Bingyang Yuan [1], Shuai Yuan [2,3] and Hao Wang [1,4,5,*]

[1] College of Shipbuilding Engineering, Harbin Engineering University, Harbin 150001, China; yuanby@hrbeu.edu.cn
[2] School of Health and Life Sciences, University of Health and Rehabilitation Sciences, Qingdao 266113, China; yuanshuai1006@hotmail.com
[3] Ocean Decade International Cooperation Center (ODCC), Qingdao 266520, China
[4] Qingdao Innovation and Development Base, Harbin Engineering University, Qingdao 266000, China
[5] Nanhai Innovation and Development Base, Harbin Engineering University, Sanya 572024, China
* Correspondence: wanghao_work@hrbeu.edu.cn

Table S1. Results of the mesh independence study at Re = 50.

Mesh scheme	Maximum mesh size [μm]	Minimum mesh size [μm]	△P [kPa]	$\frac{\lvert \Delta P_{k+1} - \Delta P_k \rvert}{\Delta P_k} \times 100\%$	M.I	$\frac{\lvert \mathrm{M.I}_{k+1} - \mathrm{M.I}_k \rvert}{\mathrm{M.I}_k} \times 100\%$
1	17	1.01	29.1	-	0.601	-
2	15.2	0.98	29.2	0.3%	0.589	2%
3	13.1	0.85	29.1	0.3%	0.607	3%
4	10	0.65	28.8	1%	0.623	2.6%
5	9	0.58	28.2	2%	0.625	0.3%
